# MetaRehabVerse: The Great Opportunity to Put the Person’s Functioning and Participation at the Center of Healthcare

**DOI:** 10.1177/15459683241309587

**Published:** 2025-01-04

**Authors:** Giovanni Morone, Irene Ciancarelli, Rocco Salvatore Calabrò, Antonio Cerasa, Marco Iosa, Francesca Gimigliano

**Affiliations:** 1 Department of Life, Health and Environmental Sciences, University of L’Aquila, L’Aquila, Italy; 2 San Raffaele Institute of Sulmona, Sulmona, Italy; 3 IRCCS Centro Neurolesi “Bonino Pulejo,” Messina, Italy; 4 Institute of BioImaging and Complex Biological Systems, Catanzaro, Italy; 5 Sant’Anna Institute, Crotone, Italy; 6 Department of Psychology, Sapienza University of Rome, Rome, Italy; 7 Scientific Institute for Research, Hospitalization and Healthcare Santa Lucia Foundation, Rome, Italy; 8 Department of Mental and Physical Health and Preventive Medicine, University of Campania Luigi Vanvitelli, Naples, Italy

**Keywords:** metaverse, neurorehabilitation, augmented reality, ICF, disability

## Abstract

**Background and Objective:**

The metaverse refers to a digital realm accessible via internet connections using virtual reality and augmented reality glasses for promoting a new era of social rehabilitation. It represents the next-generation mobile computing platform expected to see widespread utilization in the future. In the context of rehabilitation, the metaverse is envisioned as a novel approach to enhance the treatment of human functioning exploiting the “synchronized brains” potential exacerbated by social interactions in virtual scenarios.

**Results:**

The metaverse emerges as an ideal domain for adapting the principles of the—International Classification of Functioning. Its intrinsic capacity to simulate interactions within virtual environments shared by multi-users, while providing a profound sense of presence and comprehensive perception, should facilitate learning and experiential understanding. Technical and conceptual aspects are currently under definition, including the interplay with artificial intelligence, definition of social metrics performance, and the utilization of blockchain technology for economic purposes.

**Conclusion:**

Building upon these foundations, this paper explores potential areas of metaverse applications in rehabilitation and examines how they may facilitate the pillars outlined in the World Health Organization’s Rehabilitation 2030 call for action.

## Graphical abstract


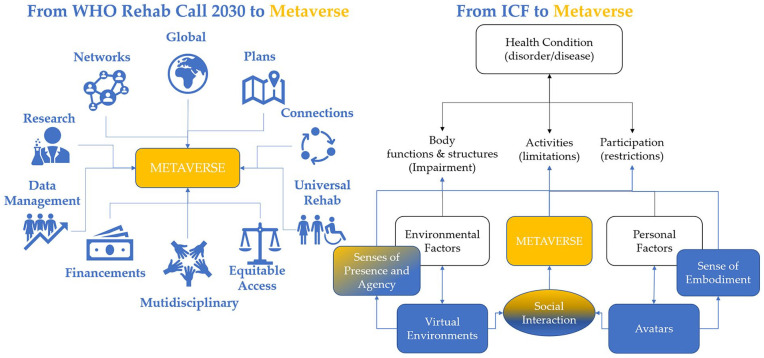


## Introduction

The metaverse is a multi-user, integrated, and interactive virtual world that has seen a surge in its possibilities across various sectors of social communication and education in recent years.^1^ A rising body of research indicates a wide range of potential uses for the Metaverse also in healthcare, including prevention, diagnosis, treatment, and cure. However, the arrival of Metaverse in the health systems brought to light a number of challenges and pitfalls that had previously been identified for tele-rehabilitation services. The main challenges, as recently described by Veras et al,^2^ might include: (i) equity (ie, accessibility and inclusivity and everyone everywhere can access quality “metaverse” healthcare services), (ii) health service integration (ie, good-quality services along the continuum of care), (iii) interoperability (ie, security and privacy), and (iv) global governance (ie, decentralization, accountability, and sustainability). On the other hand, among the pitfalls we may include the digital divide (ie, the gap between individuals, communities with or without adequate technological infrastructures, rich and under-resourced regions that have access to modern Information and Communications Technology]), and the knowledge gap between fast technology development and slow responses of healthcare providers and governments to regulate and provide standardized guidelines about the use of these new digital tools, even in the developed countries. If not consistently addressed, both these pitfalls might increase inequity in the use of new technologies in healthcare, opening important new ethical and legal issues. These problems could mainly be related to the supranational or international nature of the Metaverse that go beyond the cultural differences among countries and beyond the perspective of national legal systems. Health policy makers should also establish a legal framework to regulate the many gray areas swiftly, while scientists and clinical researchers need to grasp the potential impact of the metaverse on specific medical fields. The specific areas that require regulation are certainly patient safety, data privacy, ethical considerations, and infrastructure ownership (if private, public, or shared).

In healthcare, as indicated by available scientific literature, the metaverse can prove beneficial for several aspects, including promoting well-being and public health, conducting clinical trials, enhancing communication with health systems, alleviating anxiety and stress related to medical procedures among vulnerable populations, and enhancing medical and surgical education.^1-5^

From a rehabilitation standpoint, the metaverse holds promise in various domains, particularly due to the unique characteristics of the virtual digital universe and rehabilitation. These intersections may have significant repercussions compared to other health areas.^6,7^

Nonetheless, clinical evidence of metaverse in neurorehabilitation are at initial stage. This delay is mainly dependent upon the fact that standardized commercial solutions, which take use of the multi-user virtual scenarios of the metaverse-related device, are not available for neurorehabilitation needs due to the limitations of the actual technology. In fact, such solutions and/or platforms are meant to facilitate social interaction in virtual environments where people may meet, converse, learn, or follow lessons (ie, Unreal Engine; Microsoft Mesh; and XREngine, HyperCube). Unfortunately, these tools are not designed to provide motor or cognitive tasks that can be performed also using haptic devices for rehabilitation purposes.

For this reason, since the lack of consistent point-of-view or evidence about the application of this kind of technology in the neurorehabilitation realm, we sought to identify potential areas and aspects within this domain where the metaverse could aid in its global development. In the following sections, we present the key features of the metaverse to thoroughly assess how, why, and whether this type of technology could be used in neurorehabilitation in the future. Following 2 decades of advancement in the fields of virtual reality (VR) and tele-rehabilitation, we think that technology related to the metaverse could significantly advance personalized medicine.

References for this paper were identified through searches of PubMed with the search terms, “metaverse,” “artificial intelligence,” and “rehabilitation” until November 2024. Articles were also identified through searches of the authors’ own files. Only papers published in English were reviewed. The final reference list was generated based on originality and relevance to the broad scope of this paper.

## Metaverse and Rehabilitation

The field of rehabilitation is marked by considerable complexity, encompassing the management of disabilities of diverse origins (motor, sensory, cognitive, and communicative), collaboration with various professionals beyond medical and health sciences, operating in several settings (inpatient, outpatient, community, and home), and addressing various health areas related to prevention, care, “*quod vitam*” maintenance of recovered human functions, and participation.^8^ As concerns legal issues, the use of the metaverse for rehabilitation must comply with existing healthcare regulations, such as Health Insurance Portability and Accountability Act in the United States, General Data Protection Regulation in Europe, and other relevant local laws.^9^ However, the global and evolving nature of the metaverse could make it difficult to ensure consistent compliance across jurisdictions. Possible solutions are: (i) developing clear, transparent privacy policies to inform patients about how their data will be used, stored, and shared; (ii) developing comprehensive ethical guidelines for the use of the Metaverse, which should cover issues like patient consent, the scope of data collection, and the responsible use of artificial intelligence (AI)-driven tools; and (iii) adapting the regulatory frameworks to cover the specific challenges posed by the Metaverse.

Certainly, one of the most potentially impactful aspects of the arrival of the metaverse-related technologies in the field of rehabilitation could be the ability to facilitate the application and utilization of the biopsychosocial model of the International Classification of Functioning, Disability, and Health (ICF).^10^ The ICF, advocated by the World Health Organization (WHO), has redefined the concepts of functioning and disability across the domains of Body Functions and Structures, Activities, and Participation in social life. These domains are influenced by personal and environmental factors, which are also fundamental domains within the metaverse.^10,11^

**Figure 1.**
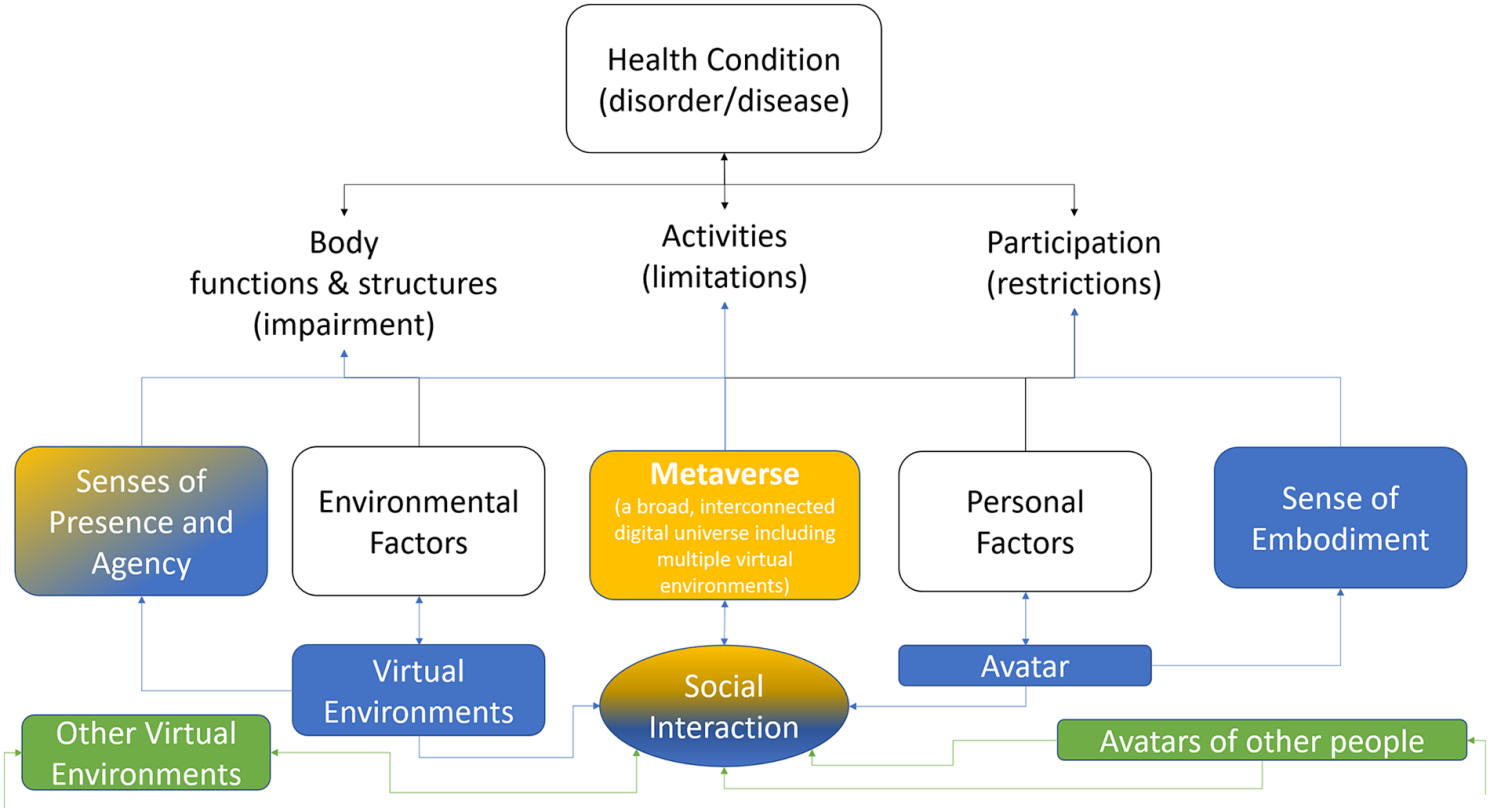
The METAREHABVERSE merging the domains of ICF (white), those of Virtual Reality (blue) also with other people or environments (green) in an interconnected digital universe (orange).

The *MetaRehabVerse* could be conceptualized as the integration of the domains of the ICF with those of the metaverse, as depicted in Figure 1. Personal factors are associated with the avatar utilized in the metaverse, thereby influencing the sense of embodiment experienced by the individual. Subsequently, the individual experiences sensations of presence and agency within the virtual environment. These sensations trigger physiological responses, as if the avatar were the individual’s real body (sense of embodiment), and the individual were physically present in a real location (sense of presence), allowing for realistic interactions with virtual objects (sense of agency).

Body representation is a multifaceted concept that is related to the perception, memory, and cognition of one’s body. The sense of embodiment in VR refers to a multidimensional experience in which the subject feels as “own” the digital body, and feels able to control and to receive sensory feedback from the virtual avatar, with the continuous presence of one’s biological body.^12^ The sense of presence allows the user to respond realistically to the virtual stimuli, eliciting physiological reactions as if the subject is physically placed in a real situation.^12^ The virtual environment supports and enables user actions that impact perception (visual, auditory, and haptic), extending the user’s sense of presence.^13^ Sense of agency is the individual perception of initiating and controlling our own actions, and ensuing perceptual consequences.^14^

The metaverse expands the 3 senses of VR, adding social interaction in a broad, interconnected digital universe of multiple virtual worlds where it is possible to interact with avatars controlled by other people or even by artificial intelligence.

The ICF is not only the tool of WHO for the classification of health and health-related domains, but it is also the framework in which the biopsychosocial model of health has been defined. In fact, the ICF conceptualizes a person’s level of functioning as a dynamic interaction between the person’s health conditions, environmental factors, and personal factors. Nowadays, many people spend a lot of working and/or spare time online, in a digital environment, using their digital identity. This redefines the boundaries of this biopsychosocial model, for the need to integrate digital life and also the new digitally-correlated health and psychological factors. From an operational point of view, the large language models of artificial intelligence that can be integrated into the Metaverse could be used in formulating ICF codes and rehabilitation prescriptions.^15^ After that, the integration between ICF and MetaRehabVerse implies to consider the principles of ICF (such as participation, social interactions, and environmental factors) also into the digital rehabilitation gym. The avatar of the patient (with or without the real physical impairment) can be together with the avatar of the therapist in a virtual environment for performing the rehabilitation session, and the therapist can select a less or more realistic digital environment, with simple or enriched tasks focused on single body functions, physical and cognitive activities, or social participation, according to the domains of ICF. If we look at the rehabilitation of the social participation, thanks to the MetaRehabVerse, we might target an unmet need of the rehabilitation: the rehabilitation of the participations. The use of the metaverse, in fact, may increase social participation with respect to conventional rehabilitation by including in the digital scenarios other patients, family members, and other people^7^ (that could also be simulated by non-playable characters and controlled by artificial intelligence). It is important to consider that people with disabilities are among the most marginalized populations in Western societies. Rehabilitation only partially responds to this enormous problem and in particular in terms of reducing impairment and increasing activity. But it does very little to promote an improvement in social participation in people with a residual and persistent disability.^16^ About this, there are psychological and behavioral problems of acceptance that could be intercepted at hospital discharge by subjecting the patient to interactions in the metaverse and mitigate, discuss potential barriers before discharge with the help of family members. The objective of this part of the rehabilitation would be to facilitate a less stigmatized outlook on disability and to create an opportunity for caregivers, researchers, and healthcare professionals to view persons with the residual post rehab disability, as whole and complex human beings, according to ICF principles.^16^

In this way we can highlight potential behavioral barriers and, for example, alteration in coping strategy regarding the illness beliefs and related emotional responses, that can lead the patient, once discharged at home, to isolate himself (reducing social participation and quality of life) and accelerating the processes of reduction of recovered functionality through non-use learning processes and maintain a healthy movement behavior.^17,18^

From the other side the integration of the Metaverse to the ICF may arise specific challenges. In particular specific digital and technical competencies should be developed in rehabilitation hospital, a major integration between hospital professionals, caregivers, should be allowed with the evaluation of subjects’ specific life environment. And obviously well-planned clinical research studies should demonstrate the transnationality of this hypothesis.

To be specific, other examples can be found in recently registered projects (on ClinicalTrials.gov). First, the Metaverse could accelerate and enhance the development of platforms for telerehabilitation (https://clinicaltrials.gov/study/NCT06354595?cond=metaverse&rank=5). In fact, the Metaverse could improve physical interactions between therapists and patients in telerehabilitation, moving from 2D video calls to avatar interactions in a 3D digital world. Another possibility is the development of specific virtual scenarios not only for physical therapy, but also for psychological and cognitive interventions (https://clinicaltrials.gov/study/NCT06418997?cond=metaverse&page=2&rank=11). Small groups of patients could interact with each other and with clinical staff in reconstructed real-life situations or perform tasks that would otherwise be impossible to carry out in hospital gyms.

Additionally, this technology could be particularly appealing to young adults, a group that is often less involved in rehabilitation. For instance, it could be used in the rehabilitation of sports injuries, helping athletes return using (virtual) playing fields sooner, and in safe, simulated sports scenarios. Another possible application for young adults is risk prevention, using this captivating technology to promote healthy nutrition, physical activity, and psychological resilience (https://clinicaltrials.gov/study/NCT05332886?cond=metaverse&rank=6). Also young adults needing sexual therapies could benefit from this technology.^19^ A recent randomized clinical trial showed that participants treated at home using a platform based on the Metaverse approach were more satisfied than those treated in clinical settings. They felt more comfortable with at-home therapy, which was more convenient, flexible, and better balanced with other commitments compared to conventional therapy. It also allowed them to avoid the anxiety and embarrassment associated with going to a clinic.^19^

The Metaverse was also used in elderly to improve motivation in case of collaborative VR-based exergaming^20^; in pediatric population with Cerebral Palsy to improve gross motor function and cardiopulmonary function^21^; and to provide innovative psychosocial support for pediatric, adolescent, and young adult subjects affected by rare cancer.^22^

Along with the potential benefits of using the Metaverse to support rehabilitation, some limitations should also be considered, particularly in the 5 main areas shown in Table 1.

**Table 1.** Metaverse and Related Rehabilitation Scenarios.

Areas of interest	Rehabilitation	Metaverse (MTV)	Strengths (S) and limitations (L) of the MetaRehabVerse
Prevention	Rehabilitation is the key health strategy for the achievement of Sustainable Developmental Goal 3 (SDG3): Ensure healthy lives and promote wellbeing for all at all ages.^8^	A metaphysical universe serves as a repository for securely stored health data (Lifelogging; primary prevention). Integrated with artificial intelligence (AI), this platform can predict the duration of disability following a minor traumatic event in frail individuals (secondary prevention).	S: The metaverse-related technologies (including AI algorithms for outcome prediction) enables the implementation of preventive rehabilitative interventions for a targeted group of individuals at risk of developing a disability. L: Challenges include potential errors in AI models and ethical concerns regarding the data used to train these models.
Continuum of care	Rehabilitation encompasses across different settings from acute Hospital care to Outpatient care within the Hospital, then extending into the Community, and finally reaching the home environment.	MTV offers independence from physical constraints such as space and time, allowing the creation of interactive and interconnected 3D worlds. Additionally, there is potential to generate a digital twin of the Rehabilitation Hospital within this digital space.	S: The metaverse facilitates personalized medicine by enabling interaction shared by multi-users with healthcare professionals throughout the Continuum of Care, spanning from the acute to chronic phases and transitioning between hospital and home settings. L: Challenges such as the Digital Divide and the necessity for caregivers with digital competencies may hinder access and utilization of metaverse-based healthcare services.
Participation	Social participation of individuals with functioning limitations is not consistently addressed throughout the rehabilitation process.	MTV enables individuals to immerse themselves in various scenarios and interact with others, thereby promoting social interactions.	S: The potential to develop interactive scenarios within the metaverse that simulate real-life situations upon reintegrating into the community, allowing individuals with new motor-cognitive abilities to interact with various people (family, friends, and strangers) and become accustomed to both others’ reactions and their own responses beyond a protected environment. L: The associated costs of designing metaverse scenarios and analyzing content.
Education	There is a necessity to strengthen the education in Rehabilitation among medical students and to implement quantity and quality programs in PRM and allied rehabilitation Health Professionals, as well as PhD programs.	Interactive 3D spaces and objects, particularly human bodies, on which to simulate interventions and assessments.	S: MetaRehabVerse would allow training on new required skills, simulating operational scenarios under stressful conditions of the systems (eg, interventional physiatry and communicating severe prognosis). L: The decrease in real interactions with patients during training might potentially lead to a poorer doctor–patient relationship in the future.
Research	There is a necessity for well-designed research studies to assess the efficacy of rehabilitative interventions. Randomized controlled trials (RCTs) and observational studies (OS) are both time and resource-intensive methods.	The potential exists to create an avatar, essentially a digital twin of the patient, on which therapeutic procedures can be tested in conjunction with AI.	S: Implementing avatars for patients could help address ethical concerns and enhance the number and speed of RCTs and/or OS. L: Challenges include potential errors in AI models and ethical dilemmas related to the data used to train these models.

## Differences Between VR and Metaverse: *The Social Brain*

One of the most prevalent misconceptions about the metaverse is its equivalence with virtual reality (VR). While they share some similarities, VR is a well-defined technology^12^ whereas the metaverse is a metaphysical universe with a less precise definition. The term “metaverse” originated from Neal Stephenson’s science fiction book “Snow Crash” in 1992, where it was described as “a computer-generated universe” projected onto goggles and earphones worn by the user. More recently, Mark Zuckerberg defined the metaverse as “an embodied internet where instead of just viewing the content, you are in it,” whereas Microsoft described it as “a persistent digital world inhabited by digital twins of people, places, and things.” It is imperative to make a concerted effort to conceptualize this universe, which is still poorly defined but distinct from VR. VR is just one of the technologies that make the metaverse accessible, which is expected to encompass much more than VR itself. It will likely redefine the internet era, social interactions, and the way we work.^1^

Over the past 15 years, VR technology has gained popularity as a valuable tool in neurorehabilitation. Research has shown that interactions between the human mind and virtual environments can enhance both motor and cognitive abilities. A recent study demonstrated improvements in upper limb motor function and manual dexterity in neurological patients with motor symptoms, such as multiple sclerosis (MS), after VR training.^23^ For instance, compared to traditional therapy, VR therapy was found to reduce fatigue in persons with MS and improve their quality of life and balance. A kinematic analysis revealed that VR training improved speed and stability of the hand-to-mouth movement more than conventional therapy (−20% and +60%, respectively).^23^ Home-based VR training has also been associated with gains in postural balance in patients with Parkinson’s disease and stroke, but not significantly more than conventional therapy (*P* = .45).^24^ In the cognitive domain, systematic and meta-analytic studies have shown the effectiveness of VR-based cognitive stimulation in older individuals, those with mild cognitive impairment, and MS patients.^25,26^ Notably, significant improvements in executive functions and memory have been observed in MS patients compared to conventional treatments.^26^

In contrast to commercial VR technologies used in neurorehabilitation, the architecture of the metaverse is more service-oriented and emphasizes social and content features facilitated through the use of digital avatars.^27^ As suggested by Matamala-Gomez et al^28^ altering individuals’ internal body representations through the experience of embodiment in a virtual body is a useful strategy for managing various clinical symptoms, including motor deficits. Therefore, the future integration of the metaverse in neurorehabilitation should explore a relatively uncharted territory in patients with brain injuries: the “social brain.”

The term “*social brain*” refers to the intricate network of brain regions and functions involved in social behaviors, interactions, and understanding. Human brains are inherently wired to navigate social environments, engage in social interactions, interpret social cues, and form relationships. The social brain hypothesis posits that over evolutionary time, human brains have evolved specialized mechanisms to support complex social behaviors essential for survival, cooperation, and communication. This concept underscores the importance of social connections in shaping brain development and function, influencing various aspects of human behavior, cognition, and emotional well-being. Studying the social brain involves investigating how these neural networks operate, develop, and adapt in response to social experiences. Understanding the social brain provides insights into human nature, social dynamics, and mental health, offering opportunities to enhance our comprehension of social behavior and improve interventions for various social and neurological conditions.^29^

VR technologies serve as the primary tools for investigating various aspects of social behaviors and cognition. In this context, the emergence of the metaverse could be seen in the realm of “Social VR tools.” Since 2013, several social VR platforms, such as VRChat, AltspaceVR, and RecRoom, have been developed and primarily applied in social psychology to reduce anxiety and loneliness.^30^ However, research on the use of social VR as a tool to improve motor and cognitive deficits in patients with brain injuries is lacking. Therefore, we believe that the advent of metaverse technologies could catalyze the development of this nascent field of study, where biomedical engineers, computer scientists, neurologists, and neuroscientists collaborate to create new platforms that can more accurately simulate social events in real life. These platforms could lead to the development of new protocols tailored to clinical needs, fostering the acquisition of new abilities.

## The innovation of the Metaverse in neurorehabilitation: AI Algorithms to Manage and Control Behavioral Performance

AI stands out as one of the most significant trends in technological discussions of the 21st century, alongside metaverse technology. These 2 technologies have the potential to complement each other, enhancing the efficiency and productivity of various processes across different fields.

In the realm of neurorehabilitation, AI-based algorithms are commonly employed for post-processing data to evaluate their predictive accuracy of outcomes. Typically, during the training phase, AI algorithms are trained to distinguish between poor and excellent performers by analyzing clinical, behavioral, and biological data collected both before treatment and upon discharge. The use of this external post-processing approach has yielded abundant evidence in recent years regarding the effectiveness of AI-based algorithms in identifying and extracting the most useful features for evaluating clinical outcomes in patients with acquired brain injury.^31^ However, these algorithms are mainly utilized as classifiers and often operate offline, rather than being integrated into medical devices to provide real-time updates on patient progress to clinicians and therapists.

Healthcare professionals can benefit from AI techniques to optimize clinical diagnosis, treatment decision-making, and data analysis.^32^ Key AI elements that enhance accuracy include learning, self-correction, and knowledge updating based on feedback, reducing the likelihood of errors in clinical evaluation and rehabilitation practice.^33^ Particularly in the rehabilitation of cognitive deficits, which involves numerous clinical decisions based on empirical knowledge, these properties of AI techniques could serve as valuable additional tools in the process.^34^ When linked to the metaverse, these techniques could facilitate online multidisciplinary team meetings and remote patient assessments.

Moreover, the Metaverse could provide simulations of real-life conditions for patient assessment, allowing clinical staff to observe or interact with patients while AI controls non-player characters, making them more autonomous, adaptable, and diverse in their reactions to patient behavior. Machine learning (ML)-based techniques in neurorehabilitation are also rapidly advancing, with implications for prognosis and treatment of various clinical disorders. Several neurorehabilitation platforms currently utilize AI-driven approaches to adapt and personalize training sessions. For example, the Neuro-World consists of smartphone games designed to test players’ selective attention and visuospatial short-term memory.^35^ These games enable patients to self-administer assessments and remotely monitor their cognitive state. The Guttman Neuropersonal Trainer includes a tele-cognitive rehabilitation platform for patients with acquired brain injury, aiming to extend neuropsychological services beyond clinical settings while improving personalization, duration, and intensity of neurorehabilitation.^36^ Similarly, the Brain Training System automatically selects and schedules cognitive training tasks based on patient cognitive profiles, with exercises’ difficulty adjusted as the patient progresses.^37^ Lastly, the NeuroAIreh@b utilizes ML algorithms to optimize the personalization and adaptability of neurorehabilitation prescriptions by combining neuropsychological assessments at baseline and during training sessions, adjusting therapy parameters based on patient performance.^38^

These existing tools provide a foundation for the next generation of AI applications in medical devices integrated with metaverse services. Customizing these tools to fit the immersive rehabilitation context could revolutionize the field, improving patient outcomes and enhancing healthcare delivery. It should be emphasized that metaverse-related technologies can also be used in the neurorehabilitative program without the assistance of AI. In the next few years, a lot of work needs to go into developing and validating competitive and cooperative social rehabilitation activities in shared virtual environments. Future applications will also benefit greatly from the introduction of AI into the metaverse, but first, some legal issues (such as copyright and intellectual property rights) must be addressed. For instance, the topic of whether AI-generated “works” should be granted intellectual property rights involves more general concerns regarding the structure of copyright. This leads to a thorough analysis that centers on 2 key questions: first, how the concepts of “free” and “protected expressions of ideas” should be interpreted in the age of generative AI, and second, whether the input and output of algorithmic processes can be legally appropriated and recognized by copyright.

## The Innovation of the Metaverse in Neurorehabilitation: Blockchain Technology Applications in Healthcare

One of the main innovations brought by the metaverse is its implementation of blockchain technologies in medicine. Blockchain is a distributed ledger system that is decentralized and records transactions across several computers in a way that prevents them from being changed later. A blockchain is a chain of secure data that consists of individual blocks that are each composed of a list of transactions, a timestamp, and a cryptographic hash of the preceding block. By doing this, data security, interoperability between different healthcare systems, integrity, and transparency are guaranteed, making it resistant to fraud and manipulation.^39^ In the clinical medicine domain, blockchain technology is being increasingly explored for its potential to enhance various aspects of healthcare delivery and data management. Some key applications include (a) electronic health records; (b) supply chain management; and (c) personalized medicine.

The primary rationale for applying blockchain technology in medicine is to overcome the concept of centralization in healthcare. Centralization is the traditional system used in the past decades, and it entails the concentration of healthcare resources, services, and decision-making within 1 or a small number of organizations, usually at the regional, national, or international organizational levels. Because centralized databases gather important data in 1 location, they are easy targets for hackers. Furthermore, this configuration frequently results in problems like single points of failure; if the system of the central authority is hacked, the entire system may be affected. Telehealth and telemedicine often suffer from centralization, increasing the risk of a single point of failure.^40^ Blockchain technology addresses such critical issues by tracking patient locations, securing remote patient–doctor consultation records, monitoring prescription drugs and medical test kits along the supply chain, and validating the qualifications of medical professionals.

The advent of the metaverse is expected to stimulate the adoption of Blockchain technology aimed at developing decentralized solutions also for neurorehabilitation services. Integrating blockchain within the metaverse offers several key advantages.^41^ Firstly, it provides a secure and transparent framework for managing digital assets, identities, and transactions within virtual environments. Blockchain’s decentralized nature ensures ownership, provenance, and scarcity of digital goods, such as virtual real estate and unique digital items. Additionally, blockchain-based smart contracts enable the creation and execution of programmable agreements, automating transactions, and interactions within the metaverse.

Furthermore, blockchain’s capability to create interoperable and portable assets allows users to carry their digital possessions across different metaverse platforms, fostering a seamless and interconnected virtual experience. The relationship between blockchain technologies and the metaverse represents a promising intersection that has the potential to redefine digital ownership, economic models, and social interactions within virtual environments. As both technologies continue to evolve, their integration is expected to shape the landscape of the metaverse, providing new avenues for innovation, creativity, and collaboration across various industries and user experiences. However, as stated by Huynh-The et al,^42^ the incorporation of blockchain technology into the future metaverse projects poses some challenges. First, the management of digital twins. Metaverse is a virtual place where people interact by means of digital twins of persons, to carry out a wide range of activities. The digital twin of a person (DToP) not only replicates a distinctive person, but also constitutes a nearly instantaneous synchronized multipresence. This means having the ability to exist in multiple places at once in the digital and physical domains. The DToP creates a complex virtual representation that closely resembles the real person. Information from many remote sensors (ie haptic devices) are used to create digital twin representations in the metaverse. For blockchain to be successfully used in digital twin applications in the metaverse, standardization, privacy, and scalability are all concerns that need to be resolved. Next, another issue regarding the blockchain applications in the metaverse refers to how to protect the highly sensitive data that AI-driven systems must produce, store, and use. The metaverse is a new technological and scientific frontier and developing AI would be challenging. In the metaverse, it is challenging to determine who owns content powered by artificial intelligence. There is no way for users to determine if they are interacting with a computer-generated avatar or a genuine person. Although this technology still needs to be developed, blockchain-based encryption may give metaverse users complete control over their data, making it easier to transfer ownership of AI consent to another company.

## Can the Metaverse Meet the Needs of Rehabilitation According to WHO Rehabilitation 2030 Call for Action?

The WHO has designated rehabilitation as the key health strategy for the next millennium^11^ and, in a historic resolution,^43^ has put forward a series of initiatives to bolster and enhance rehabilitation strategies globally. In recent years, the WHO has shifted its focus toward the functioning of individuals and their reintegration into the community, prioritizing this over pathology, thanks to the framework provided by the ICF and its robust biopsychosocial principles.^44^ Built on this foundation, rehabilitation is positioned as a fundamental health strategy for individual care, despite being a relatively young medical discipline compared to other long-standing areas. With an estimated 1 in 3 people requiring rehabilitation^45^ and significant global disparities, there is a pressing need for action.

The WHO’s Rehabilitation 2030 initiative, launched in 2017,^46^ introduced a “call for action,” rallying stakeholders toward concerted and coordinated global efforts to expand rehabilitation services. To achieve this objective, 10 priority areas and corresponding actions were identified,^47^ as shown in Table 2.

**Table 2.** Ten Priority Areas of the Rehabilitation Field From “Rehabilitation 2030, a Call for Action (WHO)” and Possible Mechanisms of Metaverse Interventions.

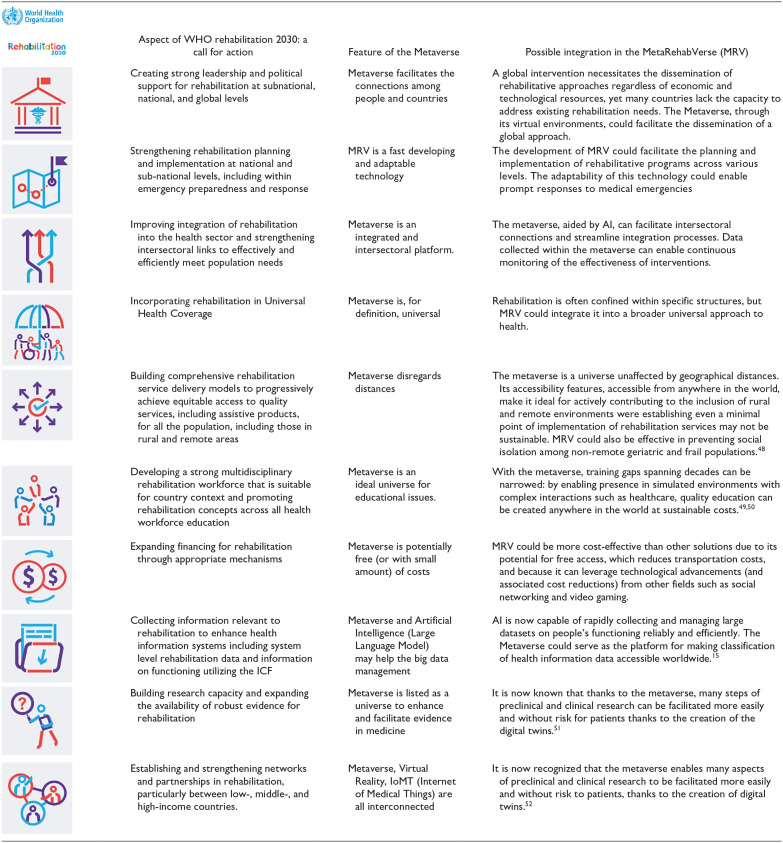

Adapted from: https://www.who.int/initiatives/rehabilitation-2030.

The *MetaRehabVerse*, if appropriately regulated and backed by significant research and development investments, could potentially support the realization of the WHO Rehabilitation 2030 call for action. Building upon the 10 priority areas outlined in the Call for Action, we hypothesize how the Metaverse could facilitate action in specific areas and why. Over the past decade, the WHO and rehabilitation stakeholders have offered technical assistance to Member States and developed a range of normative guidance and tools aimed at strengthening rehabilitation in health systems worldwide.^47,53^

Emerging technologies such as VR, augmented reality (AR), and holographic reconstruction could enhance the Metaverse, creating an integrated system where vast amounts of data from the Internet of Medical Things can be analyzed and utilized for specific purposes (eg, education, consultation, treatment, and clinical research) through human-guided artificial intelligence systems.^52^

## Discussion

Considering the social context of the Metaverse, we can propose the *MetaRehabVerse* as the cyberspace through which patients can interact and participate in collective training sessions, shifting from the individual-focused “I-rehab” perspective typical of VR treatment to a more collaborative “We-rehab” approach. This transition can significantly enhance the rehabilitation process by potentiating collective training, social interaction, and mutual support among patients. As suggested by Riva et al.,^54^ collective behaviors are founded on the acceptance of a shared frame of reference and joint action toward common goals. Communication technologies have blurred physical boundaries, enabling greater freedom in individual behavior, and digital platforms like the metaverse can facilitate information sharing, communication, and collaboration among individuals. This is why in the metaverse, patients not only may focus on the improvement of their motor and cognitive deficits thanks to the use of VR technology (ie, I-rehab), but they can also increase their social and cognitive skills by interacting with others in a safe and controlled environment (We-Rehab). Indeed, integrating the metaverse into rehabilitation holds promise for advancing toward “we-rehab” due to its accessibility and expansive nature compared to traditional VR setups. Patients can engage in therapy sessions from their homes, accessing diverse virtual environments designed to support their recovery.^54^

One of the key advantages of the metaverse in rehabilitation is its ability to foster social interaction and support networks. In fact, patients can engage in rehabilitation activities alongside others who are undergoing similar treatments. This shared experience fosters a sense of community, where patients can learn from each other’s progress, challenges, and coping strategies. Through avatar-based communication, patients can connect with peers facing similar challenges, exchanging experiences, and providing mutual encouragement, thereby enhancing motivation and adherence to rehabilitation protocols. The collaborative nature of “We-rehab” allows for the integration of gamified elements that can boost engagement, adherence to therapy and motivation. For instance, patients can participate in group challenges, working together to achieve collective goals or virtually compete in a friendly way.

The transition to “We-rehab” highlights the importance of patient-centered care, where patients may be active participants in their rehabilitation program. Thanks to the collaborative virtual sessions, patients can interact at the same time with multiple professionals (including physical therapists, speech and occupational therapists, psychologists, and several medical doctors), receive comprehensive care, and be actively involved in the decision-making process. Additionally, the metaverse empowers healthcare professionals to tailor rehabilitation programs with unprecedented precision. By leveraging data analytics and artificial intelligence, therapists can dynamically adapt virtual environments based on patient progress and feedback, ensuring that interventions remain engaging and effective throughout the recovery process.

Despite its potential, the integration of the metaverse into rehabilitation poses challenges. Technical barriers must be addressed to ensure seamless user experiences. Indeed, ensuring interoperability between different platforms, devices, and systems is a significant technical barrier. Several companies are developing their own metaverse environments, which may not be compatible with each other, leading to fragmented experiences for users. Then, there is a need to develop standards for data formats, protocols, and communication methods across platforms, such as the Application Programming Interfaces and middleware. Moreover, the different metaverse platforms and tools should be accessible to a wide range of users, including individuals with disabilities, people with different economic backgrounds, those with varying levels of technical proficiency, coming from countries different for languages, cultures, laws, economic wealth, and health systems. Among the possible solutions to overcome these issues are: (i) incorporating accessibility features from the ground up, such as voice commands, customizable interfaces, and compatibility with assistive technologies and (ii) developing low-cost VR tools that are compatible with a wide range of devices, including smartphones and older hardware.

Concerns related to data privacy, security, and ethical implications also need careful consideration to protect patient well-being within virtual environments.^9,55^ The metaverse platforms generate and handle a large amount of personal and behavioral data. Ensuring the privacy and security of this data is a major concern because of the risk of unauthorized data collection, breaches, and misuse. This is why implementing end-to-end encryption, multi-factor authentication, and blockchain technology could help secure patient data. Regular audits and updates to security measures will be crucial to staying ahead of emerging threats. Furthermore, many digital identities, each with distinct characteristics, can be created and managed by both people and AI-generated content. To faithfully capture our identities and personalities, these digital representations go beyond still photos by adding components like physical characteristics, gestures, noises, and motions. It is imperative to safeguard against the possible abuse of avatars, especially when they are used fraudulently by their creators. Because our avatars serve as extensions of our digital identities in metaverses and can affect the proprioceptive remodeling of our “self,” it is imperative that we secure them. In addition to endangering one’s reputation, this could lead to actual obligations for those involved.^55^ The preservation of personality rights is the first of several legal issues brought up by the complexity of this matter. This encompasses rights like the right to privacy, which guarantees that people are not publicly depicted without their approval, and the right of publicity, which prohibits the commercial exploitation of one’s image without authorization or payment.

Moreover, there are ethical concerns about the use of this innovative technology, mainly about the potential for exploitation or manipulation within virtual environments. This could include different issues, such as ensuring informed consent, avoiding over-reliance on virtual interactions, and preventing the misuse of therapeutic tools.^56^

Finally, the metaverse and related technologies are significantly affecting the roles, training needs, and patient interactions of healthcare professionals. While these changes can represent an opportunity for more innovative and personalized care, they also require healthcare professionals to adapt to new technologies, develop new skills, and face emerging ethical and legal challenges. In fact, healthcare professionals will need to become proficient in using advanced technologies, including VR/AR, AI, and blockchain. This could involve training in how to navigate and utilize virtual environments, operate telemedicine platforms, and interpret data generated by wearable devices and sensors. Moreover, healthcare professionals should also develop soft skills suited to digital interactions, such as building relationships with patients in virtual settings, understanding the nuances of digital communication, and managing patient anxiety or discomfort with new technologies.^56^

From an ethical point of view, it is also important to highlight that the governance of digital technologies in health care needs to be driven by public purpose and not by private profit.^57^ This is mandatory due to the tremendous impact on improving patient outcomes and access, and on changing health workers’ competencies and health workplace.^58^ In addition, a possible problem in terms of ethics should be generated from the behaviors’ health data (ie, lifelogging). This data should be acquired as behavioral health surplus data, which, if not properly regulated, can generate significant profits from privates, for example inducing health needs, and/or predict behaviors aimed at seeking health services.^59^

Incorporating the metaverse into rehabilitation represents a paradigm shift in healthcare delivery and patient engagement. Through collaboration between researchers, clinicians, and technology developers, we can fully leverage the metaverse’s potential as a tool for rehabilitation, ushering in a new era of personalized, accessible, and effective healthcare interventions.

The metaverse, with its immersive environments and potential for virtual interaction, could revolutionize rehabilitation by providing accessible, personalized care regardless of geographical barriers. Nonetheless, since there are few well-documented pilot projects demonstrating the successful integration of metaverse into clinical practice, the topic remains largely speculative in the rehabilitation field. To move beyond speculation, it would be beneficial for stakeholders to focus on developing and testing specific applications within the metaverse for rehabilitation, such as virtual therapy sessions, remote patient monitoring, or immersive training for healthcare professionals. These initiatives could then be evaluated in neurological patients (including acquired brain injury) for effectiveness, scalability, and inclusivity, providing a clearer roadmap for future integration.

## Conclusions

The implementation of a MetaRehabVerse could facilitate interaction between virtual and real cloud experts and end-users of rehabilitation content (patients, caregivers, medical operators, and healthcare managers), offering innovative opportunities for social interaction, entertainment, and new kinds of treatments based on collaborative activities in healthcare settings.

## References

[1] Kataria S Kedia AK Ravindran V. Metaverse: evolving role in healthcare delivery and implications. J R Coll Physicians Edinb. 2023;53(3):186-191. doi:10.1177/14782715231189900 37537948

[2] Veras M Labbé DR Furlano J , et al. A framework for equitable virtual rehabilitation in the metaverse era: challenges and opportunities. Front Rehabil Sci. 2023;4:1241020. doi:10.3389/fresc.2023.1241020 37691912 PMC10488814

[3] Petrigna L Musumeci G. The metaverse: a new challenge for the healthcare system: a scoping review. J Funct Morphol Kinesiol. 2022;7(3):63. doi:10.3390/jfmk7030063 36135421 PMC9501644

[4] Orr E Arbel T Levy M , et al. Virtual reality in the management of stress and anxiety disorders: a retrospective analysis of 61 people treated in the metaverse. Heliyon. 2023;9(7):e17870. doi:10.1016/j.heliyon.2023.e17870 PMC10362070 37483756

[5] Lee JH Lee TS Yoo SY , et al. Metaverse-based social skills training programme for children with autism spectrum disorder to improve social interaction ability: an open-label, single-centre, randomised controlled pilot trial. EClinicalMedicine. 2023;61:102072. doi:10.1016/j.eclinm.2023.102072 37483546 PMC10359727

[6] Cerasa A Gaggioli A Pioggia G Riva G. Metaverse in mental health: the beginning of a long history. Curr Psychiatry Rep. 2024;26(6):294-303. doi:10.1007/s11920-024-01501-8 38602624 PMC11147936

[7] Calabrò RS Cerasa A Ciancarelli I , et al. The arrival of the metaverse in neurorehabilitation: fact, fake or vision? Biomedicines. 2022;10(10):2602. doi:10.3390/biomedicines10102602 36289862 PMC9599848

[8] Morone G Paolucci S Mattia D Pichiorri F Tramontano M Iosa M. The 3Ts of the new millennium neurorehabilitation gym: therapy, technology, translationality. Expert Rev Med Devices. 2016;13(9):785-787. doi:10.1080/17434440.2016.1218275 27466820

[9] Pasa B Bernes A Gaggioli A , et al. LawVerse: the legal framework for clinical metaverse content. J Med Extend Real. 2024;1:1-14. 10.1089/jmedxr.2024.0028 PMC13393000 42558078

[10] Stucki G Kostanjsek N Ustün B Cieza A. ICF-based classification and measurement of functioning. Eur J Phys Rehabil Med. 2008;44(3):315-328. 18762741

[11] Cieza A. Rehabilitation the health strategy of the 21st century, really? Arch Phys Med Rehabil. 2019;100:2212-2214. 31128114 10.1016/j.apmr.2019.05.019

[12] Tieri G Morone G Paolucci S Iosa M. Virtual reality in cognitive and motor rehabilitation: facts, fiction and fallacies. Expert Rev Med Devices. 2018;15(2):107-117. doi:10.1080/17434440.2018.1425613 29313388

[13] Sra M Pattanaik SN. Enhancing the sense of presence in virtual reality. IEEE Comput Graph Appl. 2023;43(4):90-96. doi:10.1109/MCG.2023.3252182 37432776

[14] Braun N Debener S Spychala N , et al. The senses of agency and ownership: a review. Front Psychol. 2018;9:535. doi:10.3389/fpsyg.2018.00535 29713301 PMC5911504

[15] Zhang L Tashiro S Mukaino M Yamada S. Use of artificial intelligence large language models as a clinical tool in rehabilitation medicine: a comparative test case. J Rehabil Med. 2023;55:jrm13373. doi:10.2340/jrm.v55.13373 PMC10501385 37691497

[16] Agmon M Sa’ar A Araten-Bergman T. The person in the disabled body: a perspective on culture and personhood from the margins. Int J Equity Health. 2016;15(1):147. doi:10.1186/s12939-016-0437-2 27633249 PMC5024466

[17] de Graaf JA Wondergem R Kooijmans ECM , et al. The longitudinal association between movement behavior patterns and the course of participation up to one year after stroke. Disabil Rehabil. 2023;45(17):2787-2795. doi:10.1080/09638288.2022.2109071 35944521

[18] Della Vecchia C Préau M Carpentier C , et al. Illness beliefs and emotional responses in mildly disabled stroke survivors: a qualitative study. PLoS ONE. 2019;14(10):e0223681. doi:10.1371/journal.pone.0223681 PMC6808550 31644550

[19] Vila A Ardoy-Cuadros J Romero-Moreno R , et al. Body, emotions, and sexuality in the metaverse: a randomized control trial exploring the use of second life for an avatar-based intervention to support women with female orgasmic disorder. PsyArXiv; 2024. https://osf.io/preprints/psyarxiv/qju56

[20] Shah SHH Karlsen AST Solberg M Hameed IA . A social VR-based collaborative exergame for rehabilitation: codesign, development and user study. Virtual Real. Published online November 28, 2022. doi:10.1007/s10055-022-00721-8 PMC9702607 36465891

[21] Moon I An Y Min S Park C. Therapeutic effects of metaverse rehabilitation for cerebral palsy: a randomized controlled trial. Int J Environ Res Public Health. 2023;20(2):1578. doi:10.3390/ijerph20021578 36674332 PMC9864535

[22] Hasei J Ishida H Katayama H , et al. Utilizing the metaverse to provide innovative psychosocial support for pediatric, adolescent, and young adult patients with rare cancer. Cancers (Basel). 2024;16(15):2617. doi:10.3390/cancers16152617 39123345 PMC11311008

[23] Pau M Porta M Bertoni R Mattos FGM Cocco E Cattaneo D. Effect of immersive virtual reality training on hand-to-mouth task performance in people with multiple sclerosis: a quantitative kinematic study. Mult Scler Relat Disord. 2022;69:104455. doi:10.1016/j.msard.2022.104455 36508937

[24] Truijen S Abdullahi A Bijsterbosch D , et al. Effect of home-based virtual reality training and telerehabilitation on balance in individuals with Parkinson disease, multiple sclerosis, and stroke: a systematic review and meta-analysis. Neurol Sci. 2022;43(5):2995-3006. doi:10.1007/s10072-021-05855-2 35175439 PMC9023738

[25] Gamito P Oliveira J Alves C Santos N Coelho C Brito R. Virtual reality-based cognitive stimulation to improve cognitive functioning in community elderly: a controlled study. Cyberpsychol Behav Soc Netw. 2020;23(3):150-156. doi:10.1089/cyber.2019.0271. 32031888

[26] Maggio MG Russo M Cuzzola MF , et al. Virtual reality in multiple sclerosis rehabilitation: a review on cognitive and motor outcomes. J Clin Neurosci. 2019;65:106-111. doi:10.1016/j.jocn.2019.03.017 30898488

[27] Cerasa A Gaggioli A Marino F Riva G Pioggia G. The promise of the metaverse in mental health: the new era of MEDverse. Heliyon. 2022; 8(11):e11762. doi:10.1016/j.heliyon.2022.e11762 PMC9706139 36458297

[28] Matamala-Gomez M Maselli A Malighetti C Realdon O Mantovani F Riva G. Virtual body ownership illusions for mental health: a narrative review. J Clin Med. 2021;10(1):139. 33401596 10.3390/jcm10010139 PMC7796179

[29] Adolphs R. The social brain: neural basis of social knowledge. Annu Rev Psychol. 2009;60:693-716. doi:10.1146/annurev.psych.60.110707.163514 18771388 PMC2588649

[30] Kenyon K Kinakh V Harrison J. Social virtual reality helps to reduce feelings of loneliness and social anxiety during the Covid-19 pandemic. Sci Rep. 2023;13:19282. doi:10.1038/s41598-023-46494-1 37935718 PMC10630518

[31] Cerasa A Tartarisco G Bruschetta R , et al. Predicting outcome in patients with brain injury: differences between machine learning versus conventional statistics. Biomedicines. 2022;10(9):2267. doi:10.3390/biomedicines10092267 36140369 PMC9496389

[32] Tekkeşin Aİ. Artificial intelligence in healthcare: past, present and future. Anatol J Cardiol. 2019;22(Suppl 2):8-9. doi:10.14744/AnatolJCardiol.2019.28661 31670713

[33] Jiang F Jiang Y Zhi H , et al. Artificial intelligence in healthcare: past, present and future. Stroke Vasc Neurol. 2017;2(4):230-243. doi:10.1136/svn-2017-000101 29507784 PMC5829945

[34] Rabin LA Paolillo E Barr WB. Stability in test-usage practices of clinical neuropsychologists in the United States and Canada over a 10-year period: a follow-up survey of INS and NAN members. Arch Clin Neuropsychol. 2016;31(3):206-230. doi:10.1093/arclin/acw007 26984127

[35] Jung HT , et al. Predicting cognitive impairment level after a serious game-based therapy in chronic stroke survivors. Paper presented at: IEEE EMBS International Conference on Biomedical & Health Informatics (BHI); May 19-22, 2019; Chicago, IL; 1992:1-4. IEEE.

[36] Solana J Cáceres C García-Molina A , et al. Improving brain injury cognitive rehabilitation by personalized telerehabilitation services: Guttmann neuropersonal trainer. IEEE J Biomed Health Inform. 2015;19(1):124-131. doi:10.1109/JBHI.2014.2354537 25204001

[37] Walton CC Lampit A Boulamatsis C , et al. Design and development of the brain training system for the digital “maintain your brain” dementia prevention trial. JMIR Aging. 2019;2(1):e13135. doi:10.2196/13135 PMC6715098 31518277

[38] Faria AL Almeida Y Branco D , et al. NeuroAIreh@b: an artificial intelligence-based methodology for personalized and adaptive neurorehabilitation. Front Neurol. 2024;14:1258323. doi:10.3389/fneur.2023.1258323 38322797 PMC10846403

[39] Haleem A Javaid M Singh RP Suman R Rab S. Blockchain technology applications in healthcare: an overview. Int J Intell Netw. 2021;2:130-139.

[40] Ahmad RW Salah K Jayaraman R Yaqoob I Ellahham S Omar M. The role of blockchain technology in telehealth and telemedicine. Int J Med Inform. 2021;148:104399. doi:10.1016/j.ijmedinf.2021.104399. 33540131 PMC7842132

[41] Ali S Abdullah Armand TPT , et al. Metaverse in healthcare integrated with explainable AI and blockchain: enabling immersiveness, ensuring trust, and providing patient data security. Sensors (Basel). 2023;23(2):565. doi:10.3390/s23020565 36679361 PMC9862285

[42] Huynh-The T Gadekallu TR Wang W , et al. Blockchain for the metaverse: a review. Future Gener Comput Syst 2023;143:401-419. doi:10.1016/j.future.2023.02.008

[43] World Health Organization. Resolution: Strengthening rehabilitation in health systems; 2023. Accessed June 7, 2024. https://apps.who.int/gb/ebwha/pdf_files/EB152/B152(10)-en.pdf

[44] World Health Organization. International Classification of Functioning, Disability and Health (ICF); 2001. Accessed October 24, 2024. https://www.who.int/standards/classifications/international-classification-of-functioning-disability-and-health

[45] Cieza A Causey K Kamenov K Hanson SW Chatterji S Vos T. Global estimates of the need for rehabilitation based on the Global Burden of Disease study 2019: a systematic analysis for the Global Burden of Disease Study 2019. Lancet. 2021;396:2006-2017. doi:10.1016/S0140-6736(20)32340-0 33275908 PMC7811204

[46] World Health Organization. Rehabilitation 2030 Initiative; 2017. Accessed November 14, 2024. https://www.who.int/initiatives/rehabilitation-2030.

[47] Bernhardt J Urimubenshi G Gandhi DBC Eng JJ. Stroke rehabilitation in low-income and middle-income countries: a call to action. Lancet. 2020;396(10260):1452-1462. doi:10.1016/S0140-6736(20)31313-1 33129396

[48] Shu S Woo BKP . Pioneering the Metaverse: the role of the metaverse in an aging population. JMIR Aging. 2023;6:e40582. doi:10.2196/40582 PMC9947819 36662547

[49] Lewis KO Popov V Fatima SS. From static web to metaverse: reinventing medical education in the post-pandemic era. Ann Med. 2024;56(1):2305694. doi:10.1080/07853890.2024.2305694 38261592 PMC10810636

[50] Sandrone S. Medical education in the metaverse. Nat Med. 2022;28(12):2456-2457. doi:10.1038/s41591-022-02038-0 36385158

[51] Gruson D Greaves R Dabla P Bernardini S Gouget B Öz TK. A new door to a different world: opportunities from the metaverse and the raise of meta-medical laboratories. Clin Chem Lab Med. 2023;61(9):1567-1571. doi:10.1515/cclm-2023-0108 36855921

[52] Soliman MM Ahmed E Darwish A , et al. Artificial intelligence powered Metaverse: analysis, challenges and future perspectives. Artif Intell Rev. 2024;57(2):36. doi:10.1007/s10462-023-10641-x

[53] Seijas V Kiekens C Gimigliano F. Advancing the world health assembly’s landmark resolution on strengthening rehabilitation in health systems: unlocking the future of rehabilitation. Eur J Phys Rehabil Med. 2023;59(4):447-451. doi:10.23736/S1973-9087.23.08160-1 37695038 PMC10548396

[54] Riva G Wiederhold BK Mantovani F. Searching for the Metaverse: neuroscience of physical and digital communities. Cyberpsychol Behav Soc Netw. 2024;27(1):9-18. 37057986 10.1089/cyber.2023.0040 PMC10794843

[55] Lin J Latoschik ME. Digital body, identity and privacy in social virtual reality: a systematic review. Front Virtual Real. 2022;3:974652.

[56] Mohammadzadeh Z Shokri M Saeidnia HR , et al. Principles of digital professionalism for the metaverse in healthcare. BMC Med Inform Decis Mak. 2024;24:201. doi:10.1186/s12911-024-02607-y 39039522 PMC11265428

[57] The Lancet Digital Health. Digital technologies: a new determinant of health. Lancet Digit Health. 2021;3:e684. doi:10.1016/S2589-7500(21)00238-7 34711372

[58] Borges do Nascimento IJ Abdulazeem HM Vasanthan LT , et al. The global effect of digital health technologies on health workers’ competencies and health workplace: an umbrella review of systematic reviews and lexical-based and sentence-based meta-analysis. Lancet Digit Health. 2023;5(8):e534-e544. doi:10.1016/S2589-7500(23)00092-4 PMC10397356 37507197

[59] Zuboff S Möllers N Murakami WD Lyon D. Surveillance capitalism: an interview with Shoshana Zuboff. Surveill Soc. 2019;17(1/2):257-266. doi:10.24908/ss.v17i1/2.13238

